# Modular Design of Vacuum Systems for Lyophilization

**DOI:** 10.1021/acs.iecr.5c02756

**Published:** 2026-03-12

**Authors:** Lorenzo Stratta, Rohan P. Kadambi, Lorena Pasero, Steven J. Burcat, Richard D. Braatz, Bernhardt L. Trout, Alexander H. Slocum, Roberto Pisano

**Affiliations:** † Department of Applied Science and Technology, 19032 Politecnico di Torino, Turin 10129, Italy; ‡ Department of Chemical Engineering, 2167 Massachusetts Institute of Technology, Cambridge, Massachusetts 02139, United States; § Department of Mechanical Engineering, Massachusetts Institute of Technology, Cambridge, Massachusetts 02139, United States

## Abstract

Computational Fluid Dynamics (CFD) can be used to characterize lyophilization vacuum systems, but these simulations are typically unique to a specific geometry. This work presents a general method for predicting pressure performance by concatenating CFD and analytical models of individual components to create a vacuum system model. This method enables rapid exploration of vacuum system layouts to build custom geometry lyophilizers, which is particularly useful for novel continuous lyophilization systems. A vacuum chamber was built to validate this pressure prediction method. The simulations were then used to design a larger vacuum tunnel for continuous lyophilization that could achieve a target pressure below 10 Pa throughout its length. This tunnel was then built, and its performance matched the predictions of the component concatenation method. These results indicate that the component simulation and concatenation methods can be effectively used to predict the vacuum performance of custom geometry lyophilization systems.

## Graphical abstract

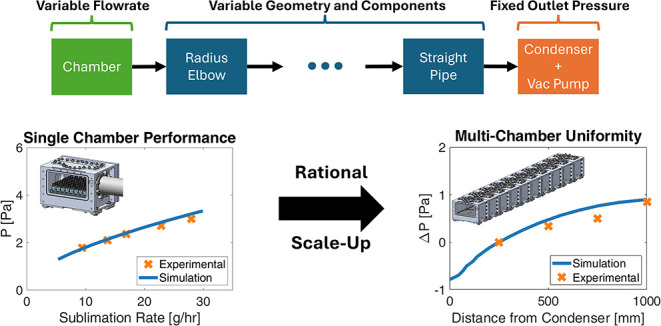

## Introduction

In vacuum freeze-drying (lyophilization), aqueous products, such as vaccines, chemotherapeutics, and monoclonal antibodies, are filled into vials, which are lyophilized in large batches within a multishelf vacuum chamber.^1^ While it can be effective at stabilizing a range of products, this process is slow because the lyophilizing product cake must remain at cryogenic temperatures throughout the drying process.^2–4^

During a typical lyophilization cycle, the liquid within the vials is frozen over a period of a few hours by cooling the shelf on which the vials sit. Then, a vacuum pump is connected to the chamber and the pressure of the system is reduced to between 5 and 30 Pa, allowing drying to occur in two stages. First, during primary drying, the bulk ice crystals are sublimated by gently heating the vials through the shelf. As this removal of water occurs, a dried, porous product is formed in the vial composed of the solids in the original solution.^4^ After all of the bulk water has been removed, typically about 95% of the original mass of water, secondary drying begins by heating the vials more aggressively to remove bound water.^5,6^ The pressure within the chamber during both steps is critical to managing the coupled heat and mass transfer that occurs within the vials.^7^

Computational fluid dynamics (CFD) modeling is frequently used within the lyophilization literature to model the process at almost every scale, from the dynamics within a single vial to the pressure uniformity within a large vacuum chamber.^8–13^ Recent developments in freeze-drying modeling include dual-scale and digital-twin approaches, which combine detailed component-level modeling with system-level predictions to support design and scale-up.^14–17^ These studies often focus on understanding, predicting, and improving the pressure uniformity within a given vacuum chamber. Additionally, simulation of different-scale vacuum chambers, such as laboratory, pilot, and industrial, is often used to aid in the scale-up of this process because the growing geometry leads to significant variation in the process.^18–20^

Industrial lyophilization requires large processing scales for this long and slow process; this operation occurs in large multishelf vacuum chambers containing thousands of vials. During typical design or scale-up, the geometry of these chambers is known, and studies can be performed to understand the large spatial variation in pressure, drying rate, or other relevant properties.^21^ The large number of vials in a chamber produces a correspondingly large sublimation rate of water that is typically managed by a single condenser. This condenser maintains inner surface temperatures below −80 °C to encourage the rapid deposition of water vapor, while a vacuum pump is used to remove noncondensable gases from passive leaks. Regeneration of these condensers, where the deposited water is removed to prepare for the next lyophilization cycle, is one of several time-intensive steps between lyophilization cycles, further increasing practical time costs.^22^

Continuous lyophilization systems have been proposed to improve process productivity and uniformity because their 24/7 operation allows them to match the average production rate of large batch vessels with smaller geometry.^22,23^ While these smaller geometries reduce the inherent spatial heterogeneity of the process, they also must be physically divided into “process zones” of different conditions, such as atmospheric and vacuum pressure. Each process zone in a continuous machine is sized based on desired productivity and process time, creating flexibility in the geometry for each machine section. Despite this enhanced flexibility and uniformity, continuous systems also introduce design challenges, particularly for stages of the process that operate under a vacuum. The system must provide airlocks to move vials in and out of the vacuum chamber and support the continuous removal of water vapor from the system through condensers. This requirement necessitates the ability to remove the accumulated ice from the condensers during operation, or multiple condensers must be used so that when one is removed for regeneration, the constant water load is still handled by another condenser.^23^ The combination of drying chamber geometry and multiple condenser installation creates a large design space for continuous lyophilization vacuum chambers, necessitating a method for rapidly exploring this space and converging with an effective design.

This work presents a design methodology for constructing the vacuum drying chamber of a continuous lyophilizer. This system was designed to maintain pressure throughout its volume below 10 Pa to ensure that sublimation occurs without cake collapse for all vials.^24^ Additionally, maintaining lower pressures limits convective heat transfer, enabling greater stability and control over the process. Because simulating the vacuum behavior of large-scale systems is computationally expensive, this work proposes an alternative method of building a library of individual component simulations and superimposing those results to predict larger system behavior. This library uses both CFD and analytical methods to predict the pressure drop in each component, leading to a prediction of the maximum pressure within the desired vacuum chamber. A small vacuum chamber was used to validate the single-component pressure-drop models before extending the methodology to the design of the larger vacuum tunnel.

## Methods

To rapidly predict pressure performance across potential vacuum system designs, a variety of components were modeled by using both analytical and CFD methods. The analytical results from Hagen–Poiseuille flow were compared to both CFD results from COMSOL and experiments performed on an equivalent geometry to determine when a full simulation is necessary for accurate results. First, a small volume chamber was built and tested to validate the modeling strategies. After validation of the overall method, the per-component results were combined with new simulations to explore the design space for a larger vacuum tunnel. Finally, this larger vacuum tunnel was also built and tested to compare its performance with simulations.

All simulations in this work were performed in COMSOL Multiphysics version 5.6. The vacuum system model uses a rarefied gas consisting primarily of water with some air, corresponding to the water vapor produced during lyophilization and air from passive chamber leaks. The low Reynolds number of the system indicated that continuum and laminar flow models were appropriate. Therefore, CFD simulations were performed using laminar compressible Navier–Stokes equations with no-slip boundary conditions applied to all solid walls. The inlet was imposed as a mass-flow (or velocity) boundary, while the outlet was defined as an area-averaged static-pressure boundary. Although the flow lies near the slip/transition regime at the lowest pressures, the characteristic dimensions of the components result in Knudsen numbers low enough that the slip velocity correction is negligible (see the Fluid Characterization section in the Supporting Information). Therefore, the no-slip formulation was adopted for the main simulations, as it provides accurate results at a lower computational cost.

### Prediction of Pressure

The overall vacuum system model consists of three categories:

1. Vapor and gas sources, such as sublimation in the vacuum chamber, that generate water and passive leaks that introduce air.
2. Vacuum piping components, which transport the vapor away from the chamber.
3. Vapor and gas sinks, such as condensers that remove water vapor and vacuum pumps that remove air.

These elements form a continuous network for the vacuum system, as shown in Figure 1, so the pressures at each component interface must be equal. Thus, modeling the pressure drop across each component is sufficient to determine the overall efficiency of vapor removal.

**Figure 1:**
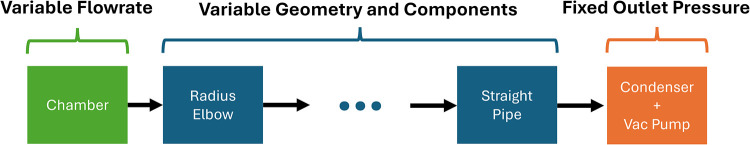
Basic vacuum system topology is simulated in this work.

The vials in the chamber are approximated as a uniform source of water vapor at the base of the chamber. The magnitude of this water vapor sublimation rate varies throughout the simulations with the target drying time and the number of vials within the chamber. This water vapor and some air from passive leaks then travel through the various vacuum components to the condenser and vacuum pump. For simulation purposes, condensers were modeled as infinite sinks of water at 0 Pa. The condensers in this study use liquid nitrogen as a cold source, maintaining a surface temperature of ≈77 K, at which the vapor pressure of water is nearly 0 Pa. Additionally, these simulations assume that vacuum pumps would be attached directly to the condensers, also providing an infinite sink for noncondensable vapors at *P* = 0 Pa under the assumption that the passive leaks are well within the pumps’ capacity. This modeling choice represents a simplifying boundary condition adopted in the absence of direct measurements of the pressure at the condenser outlet. While the partial pressure of water vapor (condensables) can be assumed negligible at liquid nitrogen temperature, noncondensable gases (inerts) are continuously evacuated by the vacuum pump. Given the good isolation of the system, the amount of inert gas introduced by passive leaks is expected to be very limited and well within the pumping capacity. As a result, the total pressure at the condenser outlet is assumed to remain very low and is weakly influenced by the pump efficiency under the operating conditions considered here. This approximation differs from that used in conventional freeze-drying systems, where inert gas is intentionally introduced to control pressure and should therefore be interpreted as a system-specific assumption.

While the vacuum chamber, condenser, and vacuum pump provide boundary conditions for the simulation, the vacuum piping components require a modeling method that captures the effects of vapor transport through them to predict viscous losses. These pressure drops can be represented as a function of the mass flow rate, geometry, and pressure boundary conditions for each component.

1 $$\Delta P_{i}=P_{up,i}-P_{dn,i}=f\left(ṁ,\;\mathrm{geometry},\;P_{up,i},\;P_{dn,i}\right)$$

The mass flow rate is determined by the lyophilization process, and the geometry is defined by the specific vacuum components. The downstream pressure starts at the condenser sink, where *P*_dn,*i*_ = 0, allowing for the calculation of the upstream pressure *P*_up,*i*_ for the first component, i.e., the one that is directly connected to the chamber (Figure 1). This value then serves as the downstream pressure for the next component, creating a repeatable process up to the vacuum chamber. This strategy allows each component to be simulated separately, reducing the computational complexity. The implicit functions in eq 1 can be estimated analytically or with CFD simulation.

#### Analytical Methods

The low system Reynolds number allows for the use of laminar pressure drop models and correlations; therefore, the Hagen–Poiseuille relation is used to create analytical models for the pressure drop across components. These solutions relate the pressure drop across a component to the flow rate and a “conductance” property (*C*_*i*_) based on component geometry,

2 $$\Delta p=\frac{\mathrm{RT}}{M_{w}}\frac{ṁ}{C_{i}\left(\Delta P,\;ṁ,\;\mathrm{geometry}\right)}$$

Analytical expressions for various components, such as straight tubes with arbitrary cross sections or elbows, are provided in the Conductance Expressions section in the Supporting Information. These functional forms allow direct implementation of the procedure in the Prediction of Pressure. Following this procedure for various geometries and connection topologies provided a rapid initial understanding of the vacuum system design space.

#### CFD Simulation

Computational simulations do not provide explicit expressions for the functional form in eq 1. Instead, the CFD results directly provide the upstream pressure for a component given the specific geometry, mass flow rate, and downstream pressure. Thus, for a given component geometry, the resultant upstream pressure can be simulated for a relevant set of mass flow rates and downstream pressures. Since CFD simulations are performed only for a discrete set of operating conditions, the exact interface pressure of every possible system configuration is not computed directly. Instead, pressure–flow relationships obtained from the component simulations are used to interpolate the expected pressure drop of each component within the explored operating range. These interpolated pressure drops are then combined using the concatenation procedure to estimate the overall system pressure distribution following the procedure in Prediction of Pressure. The key geometric components simulated for this work are summarized in Table 1; a more detailed description of the simulated components can be found in the Detailed Description of Simulated Components section in the Supporting Information.

**Table 1:** Summary of Critical Geometry Simulated

shape	length (mm)	cross section (mm)	*ṁ*_*water*_ (g/h)	*P*_dn_ (Pa)
elbow	–	*D* = 100	27–90	[0, 10]
circular pipe	500–1000	*D* = [40, 160]	4.5–180	0
square pipe	750	*s* = [100, 125]	18–180	0

### Single Chamber Validation

The results compiled through analytical and computational modeling of vacuum system components were initially validated on a small chamber with a short connection to its condenser, subjected to a series of sublimation loads. The vacuum chamber, shown in Figure 2a, had an internal volume of dimensions 250 × 125 × 250 mm. This chamber was connected to a straight pipe attached to an elbow, which, in turn, was connected to a condenser. This system layout, shown in Figure 2b, used a combination of an elbow and a straight pipe to include two different types of geometry while also providing a practical setup for the required condenser and vacuum chamber orientations. The condenser (Figure S5 and the Condenser Design section in the Supporting Information) had a cylindrical body with an internal diameter of 80 mm and a height of 325 mm. This steel condenser body was suspended in a dewar filled with liquid nitrogen to maintain the low temperature required to remove water vapor from the vacuum system.

**Figure 2:**
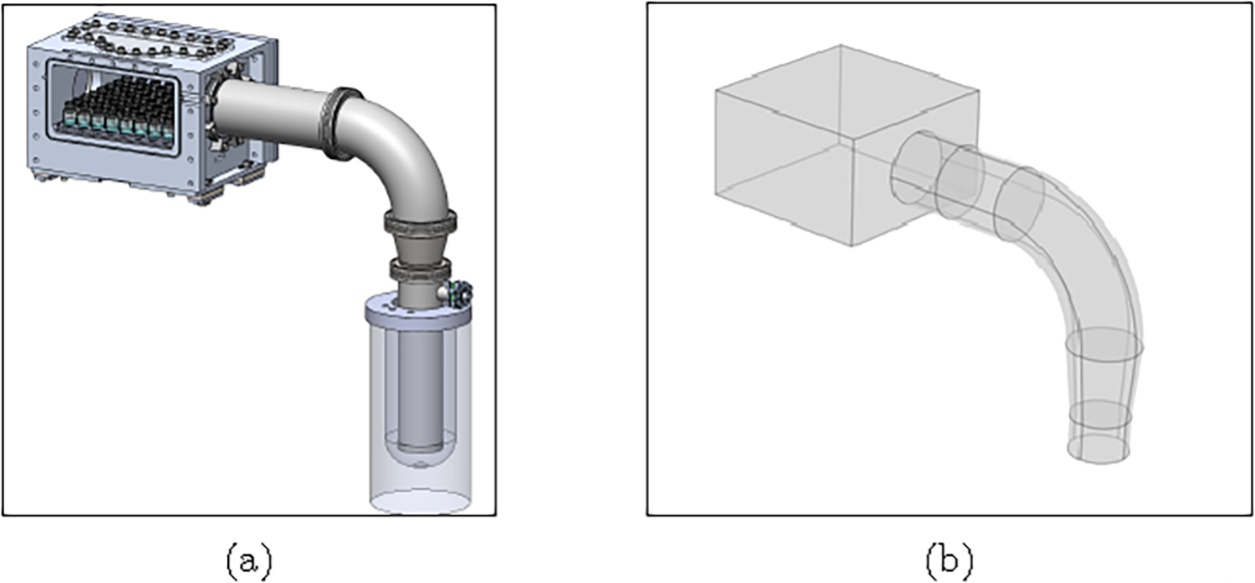
Single chamber vacuum (a) setup and (b) simulation model.

The vacuum chamber and condenser were custom-built, while the elbow and straight pipe were standard ISO100 components. The pressure was monitored with both Pirani (Agilent Varian PCG-750) and diaphragm (MKS Baratron Type 626) pressure gauges attached to the vacuum chamber, and the vacuum was pulled with an Agilent Varian DS402 vacuum pump. During water sublimation experiments, the Pirani gauges were calibrated by using the assumption that the head space was 100% water vapor. The Baratron gauge was mounted directly on the chamber outlet flange to read the chamber pressure, while the Pirani was installed downstream of the elbow on the vacuum line. Using both pressure gauges enabled simultaneous measurement of the absolute pressure and gas-composition-dependent signals during sublimation. To generate a representative sublimation of water, the chamber was filled with between 16 and 64 10R vials, each filled with 3 mL of solution containing 2.5 wt % mannitol and 2.5 wt % sucrose. These vials were frozen in a laboratory freezer at −30 °C overnight and then transferred to the vacuum chamber in an acrylic holder (Figure S4 and the Single Chamber Experiments section in the Supporting Information). During these experiments, the chamber walls were held at 25 °C and the vials were offset from the chamber base to slow heat transfer. The vials were sublimated for two h, and the total water loss was measured gravimetrically. The average sublimation rate for the vials was ≈0.4 g/(h vial), resulting in total sublimation rates between 10 and 30 g/h, depending on the number of vials sublimating.

The procedure in the Prediction of Pressure predicts the pressure only at the outlet connection for the vacuum chamber, neglecting the potential pressure variation within the chamber. The geometry of the small vacuum chamber used for the preliminary CFD validation was simulated to show that the expected pressure variation within this chamber is minimal (see the Single Chamber Experiments section in the Supporting Information). Thus, the chamber pressure variation could be neglected during preliminary validation.

It is also worth noting that inlet- and developing-flow effects can represent a significant contribution to pressure drop in vacuum ducts, particularly for the first component downstream of the chamber. For the geometry investigated here, the ISO100 duct exits from a chamber whose characteristic dimension is comparable to the duct diameter, reducing strong inlet contraction effects. However, for configurations in which a small duct exits a much larger chamber, inlet losses are expected to be more pronounced.

### Continuous Lyophilizer Tunnel Design

Once the individual component modeling was validated in the small vacuum chamber, the process was applied to design a larger lyophilization chamber. This chamber is based on an assembly of modular units that match the geometry of the small chamber, creating a tunnel. The larger lyophilization tunnel was built from ten chamber modules, creating a vacuum chamber with internal dimensions 250 × 125 × 2500 mm, as shown in Figure 3. Each of the modular chambers is designed to hold 30 vials during typical operation, resulting in a maximum sublimation flux in the tunnel of 90 g/h from vials filled with 3 g drying in 10 h.

**Figure 3:**
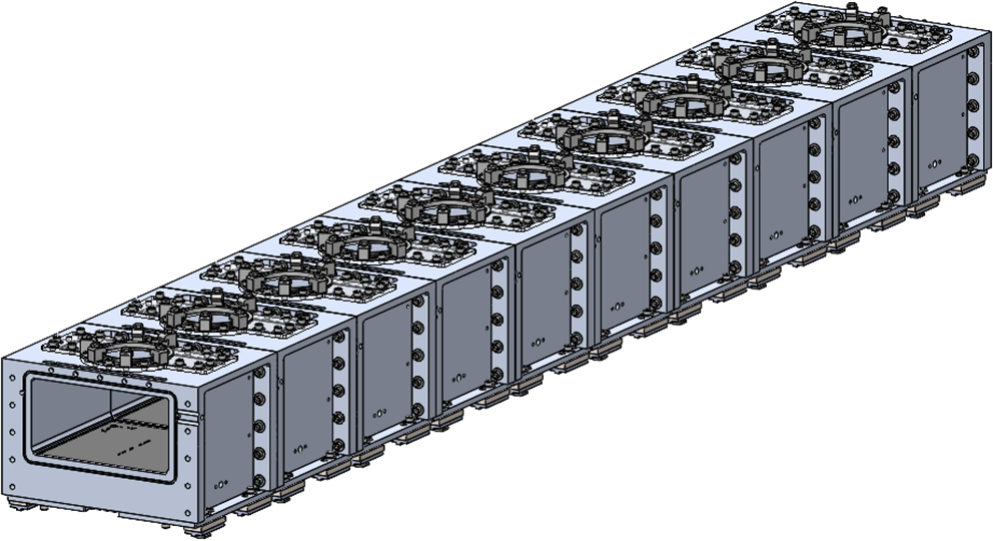
Design of the modular vacuum tunnel modeled in SolidWorks.

#### Vacuum Hardware Geometry Selection

The effect of critical dimensions, such as port diameter and pipe length, on the pressure drop was investigated to inform component selection. Port diameters were selected from ISO and KF sizes, while pipe lengths were selected as representative over a continuous range. Although larger diameters and shorter pipes are expected to reduce pressure drops, the simulations provide a quantitative analysis for the trade-off between performance and other design factors, such as geometric restrictions and cost.

#### Condenser Hookup Topology

Scaling up from the small chamber to the continuous lyophilization tunnel introduces complexity in the nonuniformity of the chamber pressure and the need for regeneration of the condensers during system operation. The larger volume and high aspect ratio lead to an expectation of a larger pressure variation in the long dimension of the tunnel compared with that of the small chamber. In addition, condensers must be separated from the vacuum environment to remove ice accumulation, while the tunnel remains under vacuum to operate the lyophilizer continuously. This regeneration requirement means that multiple condensers must be attached to the lyophilization tunnel.

The attachment topology between the condensers and the vacuum tunnel affects both the pressure uniformity and the maximum pressure within the tunnel. These condensers could either follow a direct attachment strategy, in which the condensers connect directly to ports on the vacuum tunnel (Figure 4a), or they could follow a manifold strategy, in which the condensers connect to a manifold that has more connections to the vacuum tunnel than there are condensers (Figure 4b). For both layouts, the limit of performance as the number of condensers decreases was investigated, because it minimizes hardware and because the performance of the two strategies converges in the limit of having a condenser for every vacuum tunnel port. The case of two active condensers (therefore, three total attached) was used to compare the connection strategies because it includes the complexity of multiple outlets. Here, three condensers are physically installed: two of them condensing at the same time, while the third is regenerating. The performance of each system is compared at the highest estimated sublimation rate of 90 g/h evenly distributed throughout all ten chambers because the maximum vacuum pressure is more critical to successful lyophilization than the pressure distribution within the tunnel.

**Figure 4:**
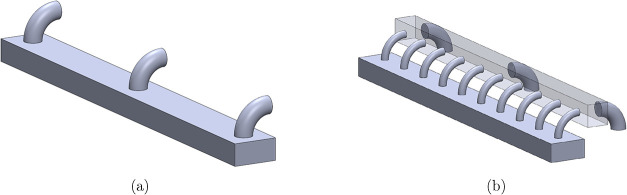
Comparison of (a) direct and (b) manifold attachment options for vacuum hardware.

An ISO100 port is the largest available outlet on each modular chamber; therefore, ISO100 elbows were used in the direct attachment simulation. In this configuration, each active condenser handles vapor generated in five consecutive chambers (the tunnel being composed of 10 chambers). The manifold strategy used a square tube with 150 mm length sides between the vacuum tunnel and equally spaced ISO100 connections for the condensers. The vacuum tunnel could be connected to the manifold either by using this same maximum available outlet diameter or by preserving the total outlet cross-sectional area, as in the direct attachment strategy. Dividing the area of the two ISO100 outlets across ten chambers results in a port diameter of 45 mm, which is close to standard KF40 and KF50 connection sizes. The latter was chosen for this simulation because the larger diameter reduces the pressure losses.

In the simulation of the direct attachment strategy, the sublimation of water was assumed to be evenly distributed across the base of the tunnel. The ISO100 elbows were assumed to have a downstream pressure of 0 Pa, approximating a connected condenser. These simulations directly estimated the pressure distribution along the long dimension of the vacuum tunnel. Analogously, simulations of the manifold strategy assumed that the sublimation rate was evenly divided between the ten KF50 inlets, and the ISO100 elbows connected to the manifold used the same *P* = 0 Pa downstream boundary condition. For the manifold strategy, the upstream pressure of each KF50 elbow was assumed to be representative of the pressure in the center of the chamber to which it was attached, and intermediate pressure values along the long dimensions of the tunnel were interpolated.

#### Integrated Tunnel and Connection Modeling

The results from measuring the different condenser topologies indicate that the direct attachment strategy provides a sufficiently uniform tunnel pressure without the hardware complexity introduced by the addition of the manifold. Using this strategy to design the specific lyophilization tunnel in this work requires predictions of the pressure response for different sublimation rates and condenser attachment layouts with more realistic boundary conditions. This tunnel must maintain a pressure below the ice-vapor equilibrium pressure to prevent cake collapse during lyophilization, plus an additional margin to provide the driving force for water vapor to diffuse out of the cake. At the collapse temperature of −34 °C for a 5 wt % aqueous sucrose solution, ice has a vapor pressure of 25 Pa. Choosing Δ*P* = 15 Pa for the driving force margin results in a maximum tunnel pressure requirement of 10 Pa.

For the 2500 mm long tunnel, a range of one to three active connected condensers was simulated (Figure 5a). The single condenser case represents the worst expected performance during operation, while two condensers are required to ensure that even when one condenser is disconnected for regeneration, the tunnel can still be serviced by a single condenser. Adding a third condenser ensures that at least two condensers are always active, providing the largest potential performance improvement for this geometry and quantifying the trade-off with adding hardware. The location of these tunnel connections affects the pressure uniformity in the tunnel, so most combinations for the 1–3 connection locations are simulated, with the exception of a single condenser in the center of the tunnel. A centrally located single condenser represents an intermediate condition between a single condenser at the edge and three symmetric condensers and would not provide additional insight into the limiting configurations relevant to the scope of this work. Because simulating the full chamber at different sublimation rates is computationally expensive, this simulation was only run for the highest sublimation rate, i.e., the worst-case scenario. The tunnel variation found in this simulation serves as a conservative estimate of the tunnel pressure variation at lower sublimation rates, such that it can be used to estimate the contribution of tunnel nonuniformity to system maximum pressure depending on the number of condenser connections.

**Figure 5:**
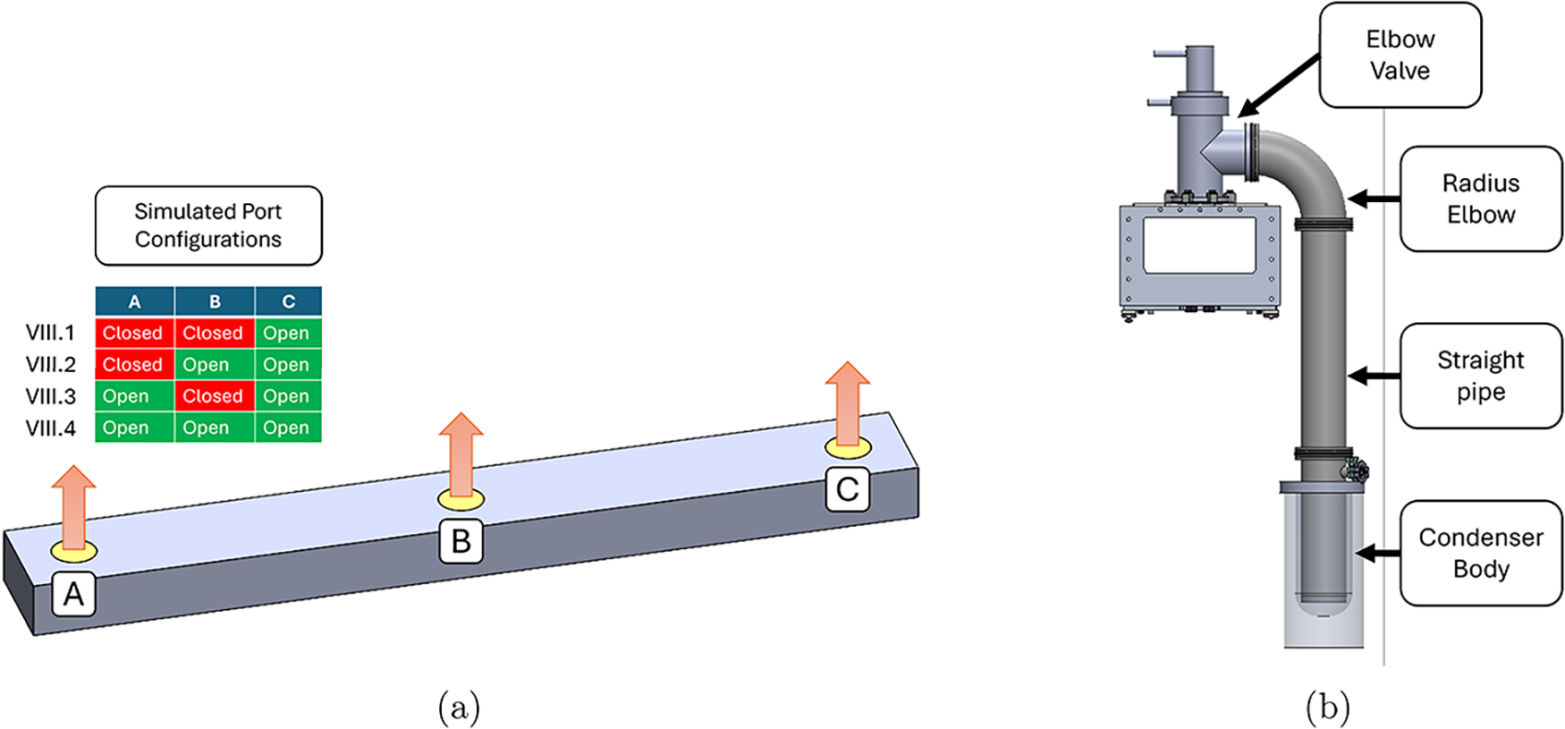
(a) Pressure distribution in the tunnel interior is simulated to compare the effects of condenser connections located at different positions along the tunnel length. This distribution is used with the pressure drop from the condenser attachment in (b) to predict the tunnel pressure for different sublimation rates and condenser attachment layouts.

For lower sublimation loads, the maximum pressure value within the tunnel was obtained by combining the component concatenation method and the simulated tunnel pressure variation. In the final apparatus, each condenser is connected to the vacuum tunnel through an identical connection branch corresponding to the geometry shown in Figure 5b. The connection geometry that was built included an ISO100 elbow valve, an ISO100 90° elbow, and an ISO100 straight pipe, which then connected to the condenser. When elbow valves are utilized, calculating the additional pressure drop is straightforward and can be derived by referencing their equivalent vacuum element, i.e., a 90° elbow. Alternative in-line options are butterfly lines, the effect of which on pressure drop was evaluated as reported in Figure S2 and the Effect of Butterfly Valve section in the Supporting Information. The results showed that butterfly valves can strongly influence choke-flow conditions and introduce significant additional pressure losses. The simulated prediction of the pressure drop along the assembly reported in Figure 5b estimates the pressure at the vacuum tunnel outlet, which represents the lowest pressure in the tunnel. The maximum tunnel pressure is then estimated by adding the conservative pressure variation value based on the condenser attachment number.

### Continuous Lyophilizer Tunnel Validation

The larger lyophilization tunnel, shown in Figure 6, was assembled by bolting chamber modules together with their interfaces sealed by O-rings. The results from simulating potential pipe lengths combined with elbow and tunnel geometries were used to design the vacuum pipe system between the vacuum tunnel and the condensers. This system uses larger (100 mm) diameter condensers than the single chamber experiments because the simulation results for different pipe diameters showed a significant benefit from using larger diameter components. The outlet of each condenser was connected through a KF25 elbow valve and corresponding piping to a single vacuum pump, allowing one pump to service multiple condensers. The pressure in this larger system was measured using Inficon PSG500 Pirani gauges in addition to the Pirani and capacitance gauges used for the single chamber. These pressure gauges were moved between multiple positions on the vacuum tunnel to determine the pressure profile throughout the tunnel. Overall, three Pirani and two Baratron gauges were distributed along the tunnel (Figure S9 and the Tunnel Experiments section in the Supporting Information). Baratron sensors ensured accurate absolute reference measurements at key nodes, while the additional Pirani gauges allowed tracking of water-vapor content and composition-dependent variations along the tunnel. Both sensor types were used to detect whether pressure differences originated from viscous losses or from vapor accumulation effects, which is essential for validating the tunnel vacuum transport behavior and condenser loading. In this vacuum tunnel, restrictions related to continuous operation limited the vial density to 30 vials per chamber module. Generating a representative water load for this entire volume would require hundreds of vials, which is impractical for validation testing. Thus, an equivalent sublimation rate load was generated using 50 mL Falcon tubes filled with ice. In these experiments, a variable number of falcon tubes were placed in the tunnel and allowed to sublimate for two hours, and their mass loss was measured gravimetrically to infer a sublimation rate. A range of sublimation rates between 10 and 90 g/h was used to represent loads from vials in one to ten full chamber modules drying over a period of 10 h.

**Figure 6:**
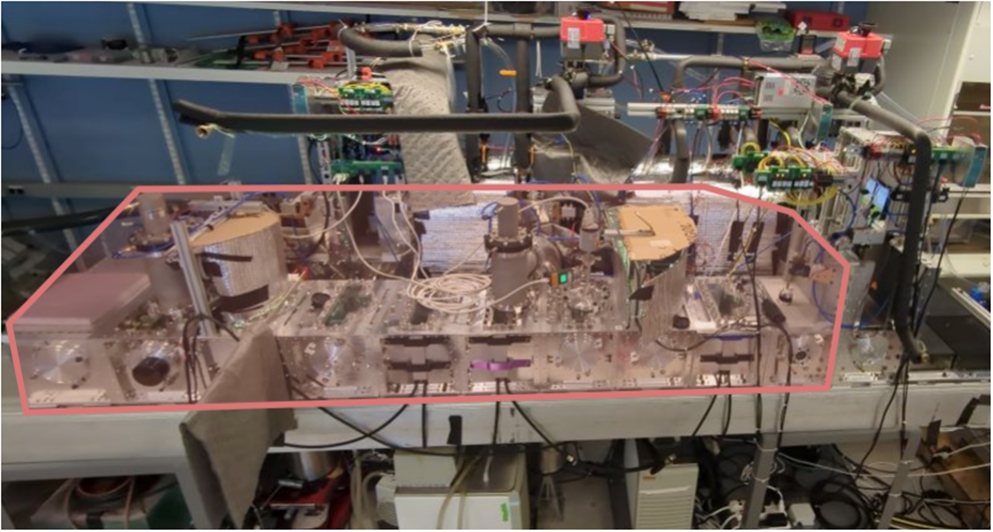
Assembled vacuum tunnel as part of a larger continuous lyophilizer.

## Results

These results show the simulation method of concatenating individual component simulations into a larger vacuum system, a comparison to pressure data collected in a small chamber system, the design of a longer vacuum tunnel for continuous lyophilization using this concatenation method, and the validation of the modeled performance of the manufactured vacuum tunnel.

### Analysis of Individual Vacuum Piping Components

The predicted pressure drops across a representative straight tube with circular cross-section (Figure 7) show general agreement as the sublimation rate changes. The model predictions for simple tube geometry agree at low sublimation rates but begin to diverge as the sublimation rate increases above 10 g/h, likely due to larger deviations from *P̅* throughout the geometry. This difference in estimated pressure remains less than 1.5 Pa for large diameter pipes with sublimation rates below 100 g/h, showing reasonably good agreement over a large range of values. However, this agreement does not extend to the 90° elbow, where the Hagen–Poiseuille predictions are ≈2× larger than the CFD simulations. This limitation in the Hagen–Poiseuille model originates from the fit parameter *K* in the conductance expression (see Table S1 and the Conductance Expressions section in the Supporting Information), which is not exact for all operating conditions.

**Figure 7:**
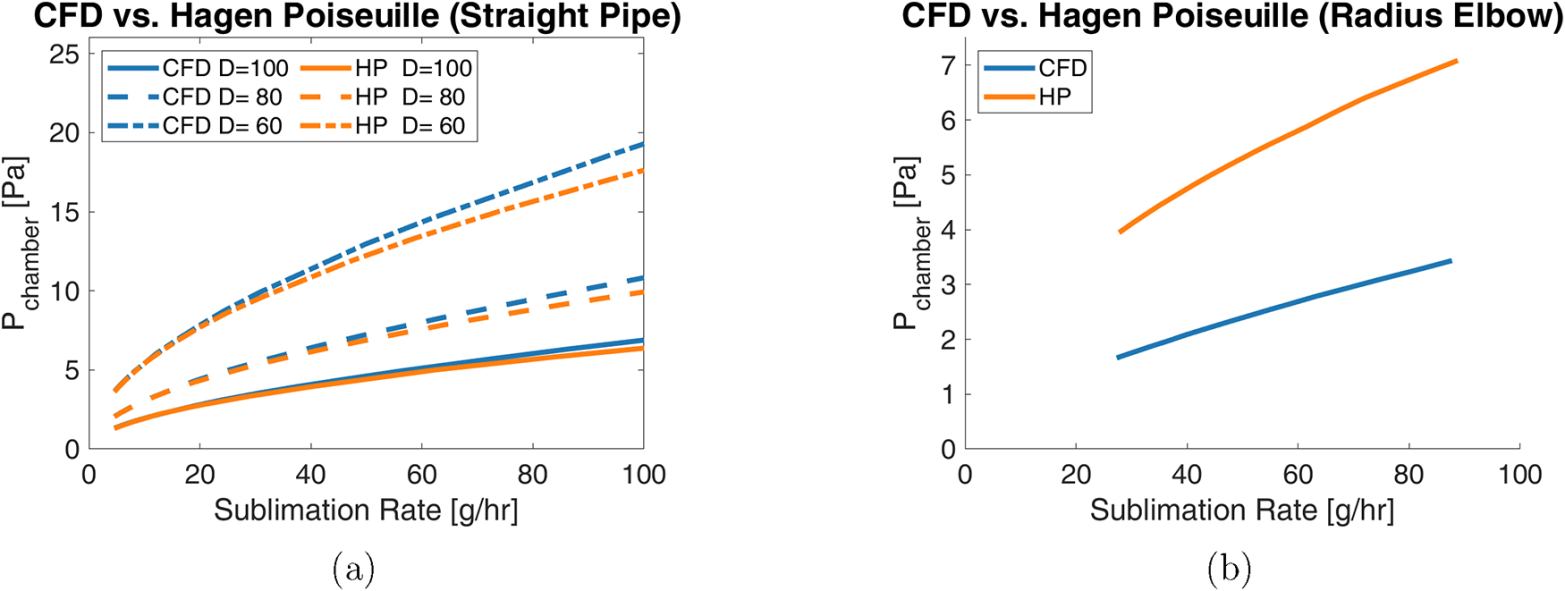
Comparison of pressure drop given a range of sublimation rates for (a) various straight pipes and (b) an ISO100 radius elbow when *P*_dn_ = 0 Pa.

While analytical solutions can reasonably predict vacuum behavior across a straight pipe, the CFD simulation is expected to be more accurate for the more complex elbow geometry. Thus, simulations were used to predict the performance of the elbow given a range of outlet pressures representative of different potential elbow positions and conditions in a larger vacuum piping system. These simulations, shown in Figure 8, indicate lower pressure drops than predicted by analytical Hagen–Poiseuille correlations at low flow rates that diminish as the flow rate increases. Consequently, components such as the elbows that redirect flow should be minimized to achieve low system pressures, and if used with straight lengths, the elbows should be positioned farther from the condensers.

**Figure 8:**
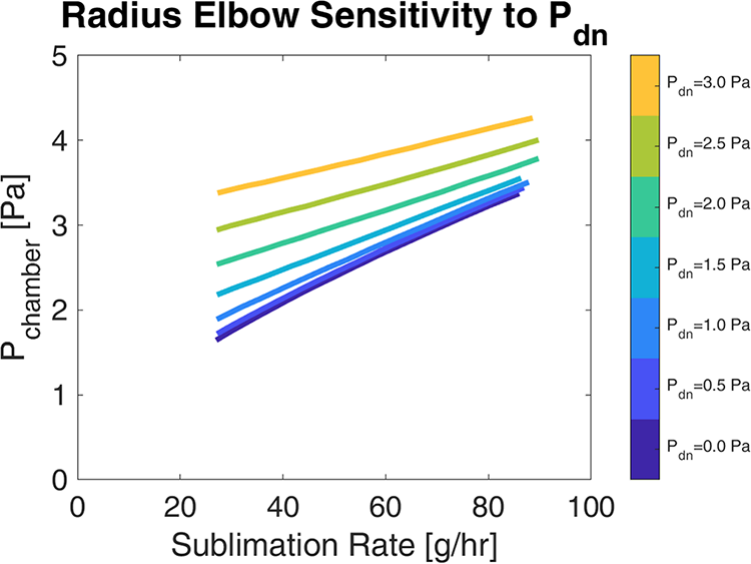
Simulated upstream pressure response of a 90° ISO100 radius elbow subject to variable sublimation rates and downstream pressure.

The full performance library for individual vacuum piping component performance for the geometry and pressure conditions described in Prediction of Pressure appears in the Detailed Description of Simulated Components section in the Supporting Information. This library was generated using CFD simulations because they are expected to be more accurate across all geometries, although the Hagen–Poiseuille analysis could reasonably extend the library for straight pipes with low sublimation rates. The results presented here are illustrative of the broader trends and comparisons throughout the component library.

### Single Chamber Results

While the components connecting the vacuum chamber to the condenser are explicit, the exact path to the water vapor sink is not as well-defined. During lyophilization experiments, the deposition of water vapor in the condensers results in the evaporation of liquid nitrogen and a reduction in the liquid nitrogen level. This phenomenon causes the location of the *P* = 0 Pa condition, where liquid nitrogen contacts the condenser body, to move. As shown in Figure 9a, the length of the condenser body above this level serves as an additional straight pipe whose length varies during operation with the changing liquid nitrogen level. Figure 9b shows the uncontrolled pressure change from a constant sublimation rate without refilling the liquid nitrogen in the condenser (see the Single Chamber Experiments section in the Supporting Information). This slow drift is well matched by the simulated pressure change based on an increasing straight pipe length resulting from liquid nitrogen evaporation. During full lyophilization experiments, the liquid nitrogen required multiple refills; therefore, its changing level was not explicitly modeled. Instead, the expected performance is bounded by the full and half-full condenser models.

**Figure 9:**
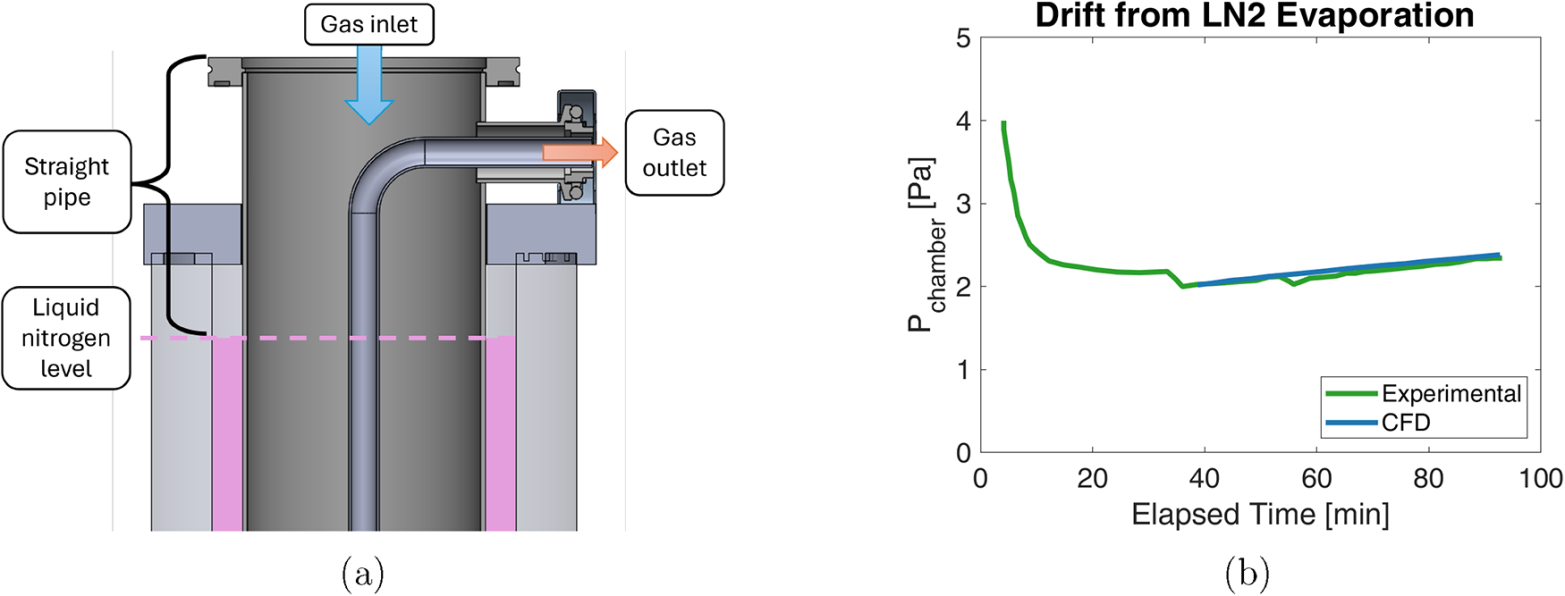
Effective length the water vapor must travel varies as the liquid nitrogen fill level shown in panel (a) changes. (b) This variation causes a measurable increase in the chamber pressure that is consistent between simulation and experimental data.

Hagen–Poiseuille models for the small vacuum chamber, straight pipe, and 90° elbow were concatenated to generate pressure drop predictions between the condenser and the vacuum chamber, while the combined geometry was simulated in CFD. These results, presented in Figure 10, show that while Hagen–Poiseuille overestimates the measured chamber pressure, the CFD simulation accurately predicts the pressure drop through the system. This Hagen–Poiseuille overestimation is expected based on the presence of the elbow, which was shown in the analysis of individual components to have an overestimated analytical pressure contribution. Thus, CFD simulations were used to guide the design of the larger vacuum system.

**Figure 10:**
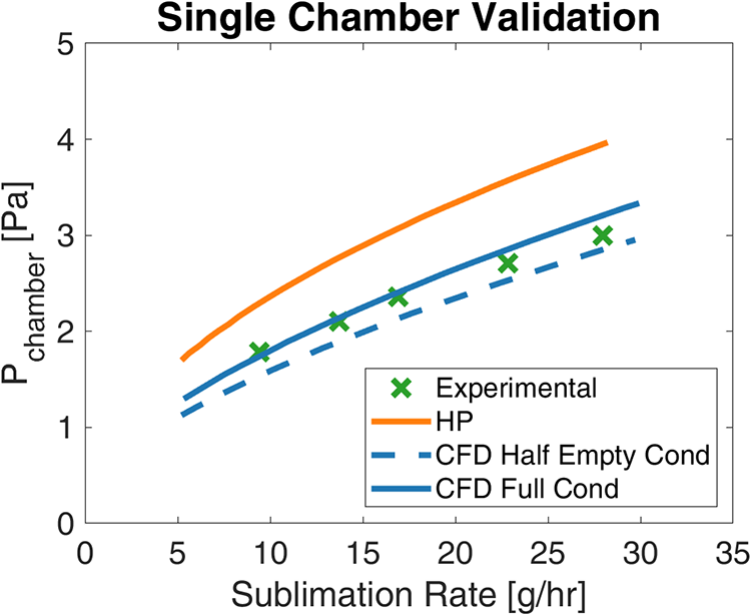
Comparison of the modeled, simulated, and measured chamber pressures within the small vacuum chamber.

### Selection of Tunnel Geometry

After the analytical model and predictive capacity of CFD simulation were evaluated with the small vacuum chamber, a wider set of geometries was simulated to explore the design space and build a larger lyophilization tunnel.

#### General Scaling

The initial simulation results provide some expected guidance regarding the length and cross-sectional area of the vacuum piping components. In general, using larger cross-sectional pipe areas and shorter pipes reduces the pressure drop across the system, resulting in a lower pressure in the vacuum tunnel. Figure 11 highlights this trend, demonstrating that larger diameter piping allows for much longer pipe lengths while maintaining low pressures. Given the tunnel wall height restriction of 125 mm, the nominal ISO100 size is selected because it is the largest standard size that does not exceed the wall height. The pressure drop is particularly sensitive at high sublimation rates to the length of piping between the vapor source and the sinks; therefore, this length is minimized within the physical restrictions of the larger system construction.

**Figure 11:**
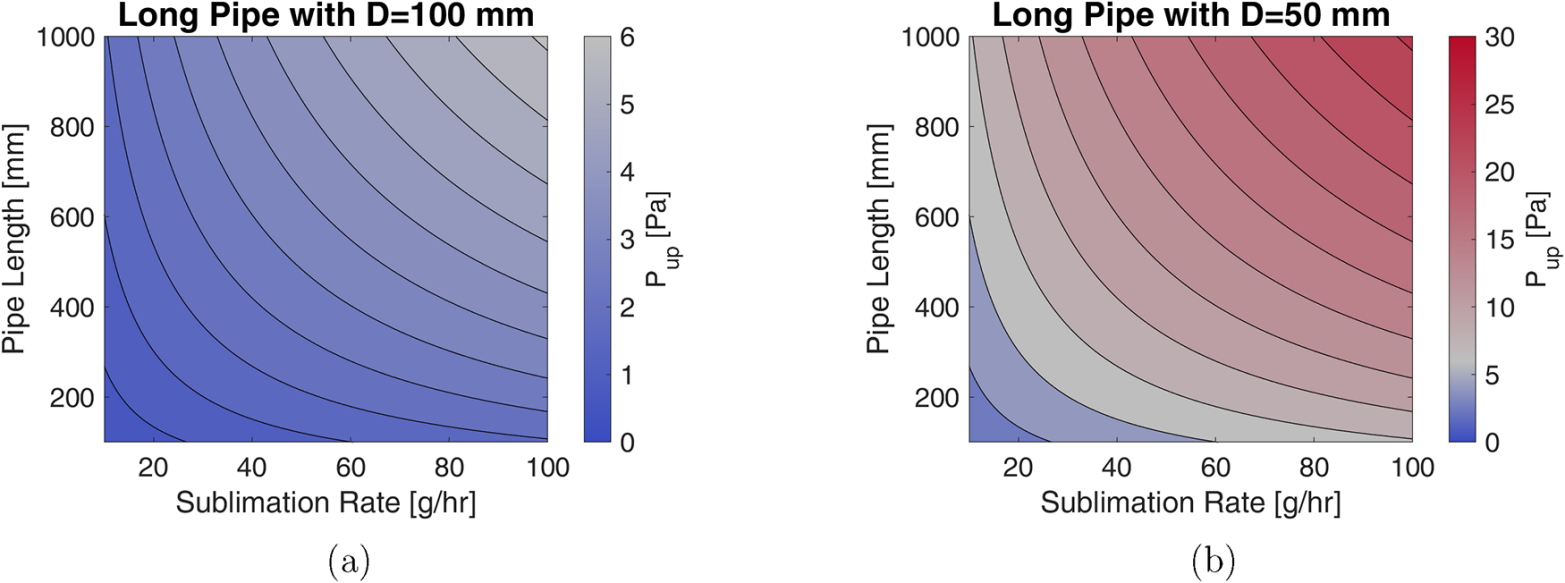
Upstream pressure variation for variable length simple pipes (*P*_*dn*_ = 0 Pa) with (a) a large diameter (*D* = 100 mm) and (b) a small diameter (*D* = 50 mm).

#### Condenser Topology

The comparison in Figure 12 of the manifold and the direct connection strategies shows that the manifold provides greater uniformity at the cost of a slightly higher average pressure. For the sake of clarity, the full set of tested configurations, including those not shown in Figure 12, is presented in Figure 13. Moreover, additional results of manifold simulations are provided in the Manifold Simulation Results section in the Supporting Information. In particular, the performance of the manifold is very sensitive to the size of the connection from the chamber with KF50 and ISO100 elbows, resulting in typical pressures of 5.8 and 3.7 Pa, respectively. Both direct attachment simulations achieve pressures lower than the ISO100 manifold, with average pressures for the (110) and (101) configurations of 3.2 and 3.3 Pa. However, while both manifold simulations result in excellent uniformity (Δ*P <* 0.1 Pa) across the tunnel, the direct attachment simulations show variation between 1.5 and 2.5 Pa.

**Figure 12:**
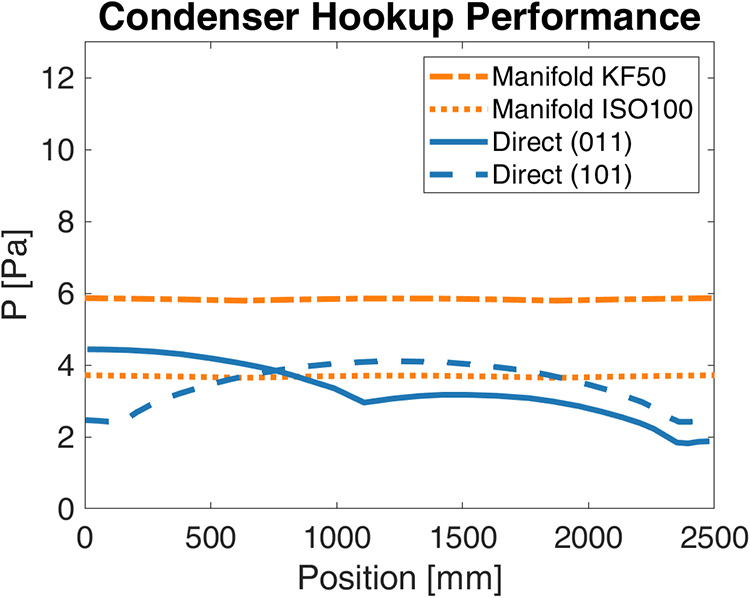
Comparison of the pressure profile within the tunnel when 90 g/h of sublimating water is managed by two condensers in the manifold (orange) and direct attach (blue) simulations. The pressure valleys along the axial coordinate coincide with the condenser direct connection locations. The plotted pressure distributions already account for the pressure losses in the connections between the chamber and the condenser.

**Figure 13:**
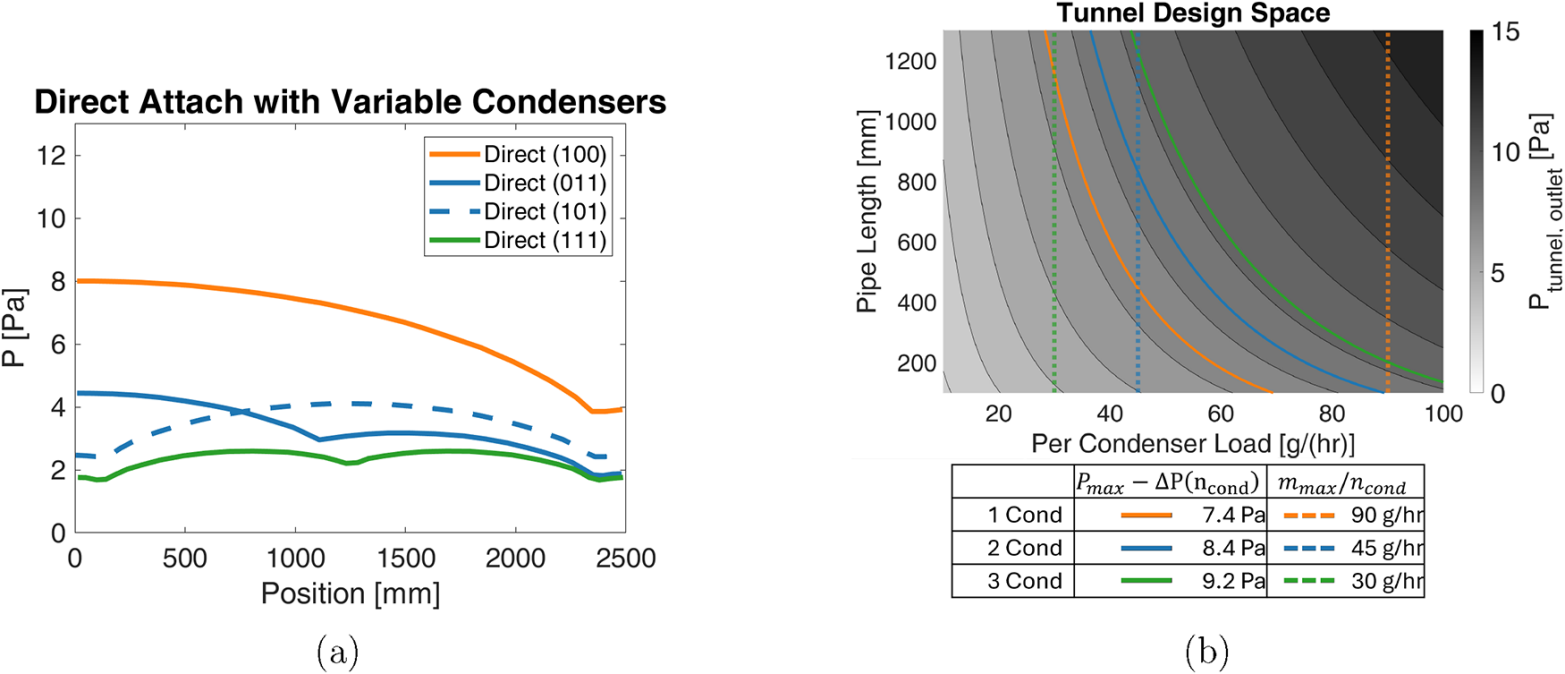
(a) Comparison of the pressure uniformity within the tunnel when one to three direct attachments are used. (b) The expected pressure within the tunnel at variable pipe lengths between the condenser and the tunnel over a range of sublimation loads. The variation in panel (a) shows the effect of the number of active condenser connections on the tunnel pressure distribution. The pressure valleys along the axial coordinate coincide with the condenser connection locations. Panel (b) demonstrates that increasing the number of active condensers reduces the sublimation rate through each. The plotted pressure distributions already account for the pressure losses in the connections between the chamber and the condenser.

The manifold configuration with KF50 elbows was excluded from further consideration because its performance was 2 Pa worse than that of the other solutions. Therefore, the decision between manifold or direct attachment configurations with ISO100 connections is a trade-off between cost, complexity, and uniformity. For this lyophilization system, the improvement in pressure uniformity was not considered sufficient to offset the additional economic and assembly costs of requiring four times as many ISO100 elbows with corresponding seal paths and custom manufacturing the manifold. Thus, the direct attachment strategy is investigated in further detail for the final system design.

#### Integrated Tunnel and Connection Modeling Results

After the direct attachment method was selected, the efficacy of this method was simulated when more or fewer condensers were attached, with the results presented in Figure 13a. Details on the simulation approach are given in the Accuracy of component concatenation section in the Supporting Information. When only one condenser is connected to the vacuum tunnel, the maximum pressure increases to 8 Pa at the point in the tunnel farthest from this connection. Using two condenser attachments nearly halves this maximum pressure to 4.5 Pa. This maximum pressure varies by <0.5 Pa for different arrangements of these two connections out of three evenly spaced ports. When all three ports are connected to condensers, the maximum pressure drops below 3 Pa. These results demonstrate that it is significantly beneficial to design for three condenser attachments such that at least two condensers remain active at all times, while the performance with three condensers is achieved when no condensers are being actively regenerated.

Additionally, the tunnel variation found in Figure 13a serves as a conservative estimate of the tunnel pressure variation at lower sublimation rates. These results can be used for a condenser number of at least one condenser per 2500 mm of tunnel length, since that was the sparsest condenser number simulated. For a condenser number greater than one condenser per 750 mm, the maximum pressure variation is expected to be less than 1 Pa; therefore, it can be neglected.

Based on external constraints of the other elements of the continuous lyophilizer, the shortest path between the vacuum tunnel and a condenser that maintains the necessary orientations of the components requires a 180° turn and a straight pipe length, leading to the assembly shown in Figure 5b. The predicted pressure using two concatenated radius elbow simulations for varied sublimation loads combined with a range of straight pipe lengths, shown in Figure 13b, is used to ensure that the available pipe length can achieve the target *P*_tunnel_ < 10 Pa. With a total load of up to 90 g/h, a single condenser would not be able to maintain the required pressure when considering the additional variation in tunnel pressure shown in Figure 13a. With two active condensers, the load per condenser and the additional variation in tunnel pressure are halved, allowing for any pipe length below 800 mm to achieve the design target. A nominal pipe length of 500 mm was selected to allow for the placement of the condensers near the floor below the lyophilizer, which reduces potential nitrogen overflow or spill risks.

### Validation of Tunnel Geometry

The tunnel described in Continuous Lyophilizer Tunnel Validation was constructed based on the results from the small chamber and further modeling. While geometrical constraints prevented the physical system from matching the condenser placement of the simulation exactly (see the Tunnel Experiments section in the Supporting Information), the predicted and experimental pressure distributions can be compared directly if the distance from a condenser is used in lieu of absolute position. Figure 14a shows that the experimental tunnel’s uniformity slightly exceeds the prediction from the simulation. Furthermore, the results from the tunnel design space calculation (Figure 13b) can be compared to the measured pressure response of the system by plotting the expected pressure at the known true pipe length (500 mm). These results (Figure 14b) show a similar shape, with the simulation slightly overpredicting the expected tunnel pressure. Overall, there is an excellent match between the simulation and experimental results, with the two matching within a margin of 1.5 Pa.

**Figure 14:**
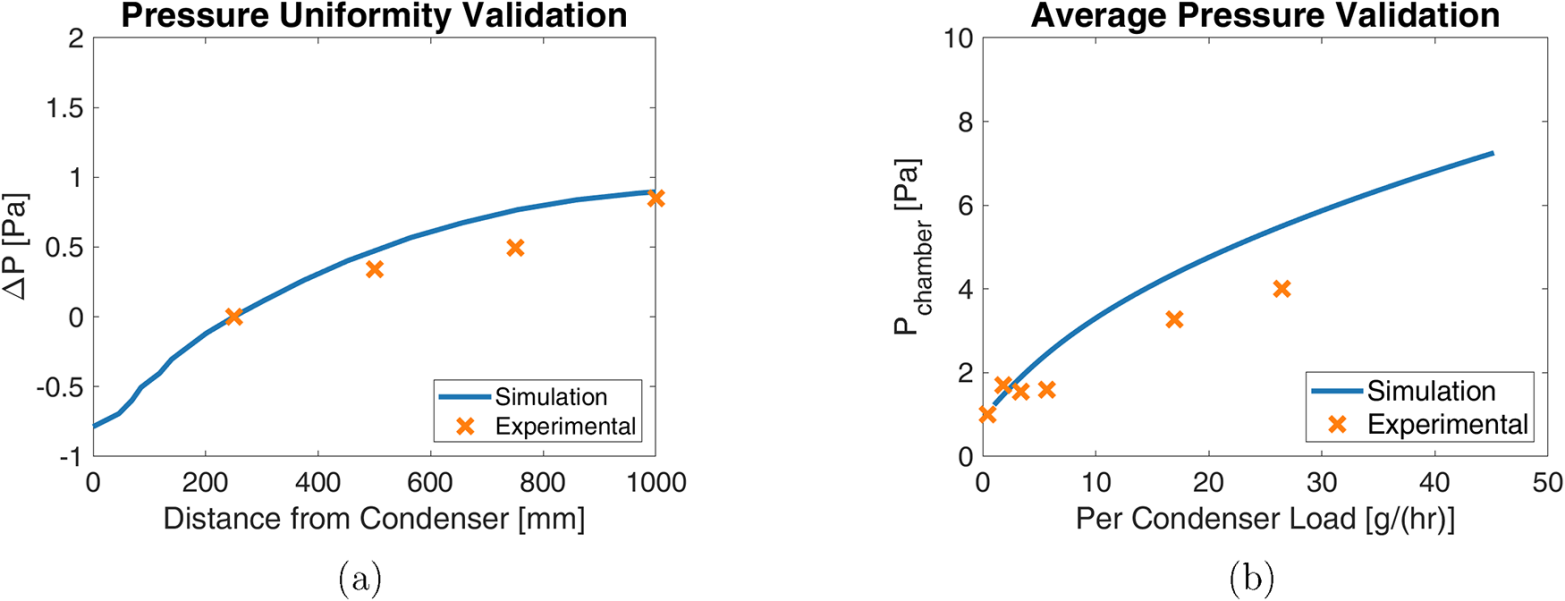
(a) Simulated pressure distribution and (b) pressure sensitivity to changing sublimation loads match the experimental data well.

## Conclusions

The individual component modeling and simulations show a high dependency of the pressure drop on pipe diameter, indicating that using larger diameter vacuum piping is more influential than maintaining short lines. In building a component performance library, Hagen–Poiseuille is effective for straight pipes, particularly at low flow rates, but simulation is required for more complex geometries, such as elbows, because Hagen–Poiseuille significantly overestimates the pressure drop. Moreover, simulation results also indicate that in-line butterfly valves can strongly influence choked-flow conditions and introduce significant additional pressure losses compared with elbow valves. Experimental results demonstrate that the component concatenation method is highly effective in predicting vacuum system performance, even accounting for variations such as changes in the liquid nitrogen level in condensers. Furthermore, these results are consistent for both a small chamber and a larger vacuum tunnel, both of which were built and tested in this study. This vacuum system component library and concatenation method for designing larger systems show promise for designing continuous lyophilization drying chambers.

## Supplementary Material



## Data Availability

The data underlying this study are available in the published article and its Supporting Information. Further inquiries can be directed to the corresponding author.

## Notes

Fluid characterization, conductance expressions, detailed description of simulated components, effect of butterfly valve, single chamber experiments, condenser design, tunnel simulation details, tunnel experiments (PDF)

## List of Symbols

*P*: pressure (Pa)
*P*_up_: upstream pressure of a component (Pa)
*P*_dn_: downstream pressure of a component (Pa)
*P*_tunnel_: pressure inside the vacuum tunnel (Pa)
*P*_out_: outlet boundary pressure (Pa)
*P̅*: area-averaged pressure (Pa)
η: efficiency (−)
*PW*_LN_2__: power delivered to liquid nitrogen (W)
*PW*_loss_: power delivered to LN_2_ through heat losses through the dewar (W)
*t*: time (s)
*ṁ*: mass flow rate (kg/s or g/h)
*C*: vacuum conductance of a component (m^3^/s)
*T*: temperature (K)
*M*_w_: molar mass of water vapor (kg/mol)
μ: dynamic viscosity (Pa·s)
*D*: pipe diameter (m)
*L*: component length (m)
*r*: elbow radius (m)
*a*,*b*: rectangular duct side lengths (m)
*S*: free surface area of LN_2_ (m^2^)
*z*: LN_2_ level inside condenser (m)
θ: elbow angle (degrees)
λ_w_: mean free path of the water vapor (m)
Δ*H*_cond,w_: latent heat of water condensation (J/kg)
Δ*H*_*v*,LN_2__: latent heat of LN_2_ vaporization (J/kg)
*K*: empirical factor for elbow conductance
*d*_w_: collisional diameter of water (m)
*N*_*A*_: avogadro’s number (mol^–1^)
