# Supplementary material for: Modular Design of Vacuum Systems for Lyophilization
Source: Ind Eng Chem Res. 2026 Mar 12;65(11):6066–76. doi: 10.1021/acs.iecr.5c02756 (PMC13022806; doi:10.1021/acs.iecr.5c02756)
Supplement: Supplementary file 1 [file ie5c02756_si_001.pdf]

Supporting Information
Modular Design of Vacuum Systems for Lyophilization

Lorenzo Stratta,[†,§] Rohan P. Kadambi,[‡,§] Lorena Pasero,[†] Steven J. Burcat,[¶] Richard D. Braatz,[‡] Bernhardt L. Trout,[‡] Alexander H. Slocum,[¶] and Roberto Pisano[*,†]

†*Department of Applied Science and Technology, Politecnico di Torino, Turin, 10129, Italy*

‡*Department of Chemical Engineering, Massachusetts Institute of Technology, Cambridge, MA 02139, USA*

¶*Department of Mechanical Engineering, Massachusetts Institute of Technology, Cambridge, MA 02139, USA*

§*These authors contribute equally to this work*

E-mail: roberto.pisano@polito.it

Fluid Characterization

Lyophilization occurs at low pressures, allowing water vapor to be approximated as an ideal gas with its density given by

$$\rho_w = \frac{M_w P}{RT} \tag{1}$$

where M_w is the molecular weight of water, P is the absolute pressure in the chamber, R is the gas constant, and T is the gas temperature. The gas viscosity can be derived from the kinetic theory of gases,

$$\eta_w = \frac{2}{3\sqrt{\pi}} \frac{\sqrt{M_w RT}}{\pi d_w^2 N_A} \tag{2}$$

where d_w is the collisional diameter of water, approximated as 280 pm, and N_A is Avogadro's number. The kinetic theory of gases can also be used to calculate the mean free path of the water vapor,

$$\lambda_w = \frac{RT}{\pi\sqrt{2} d_w^2 N_A P} \tag{3}$$

Evaluating Equations 1–3 requires a value for the temperature of the gas. Given the low pressure conditions and relatively low contact area between the vapor and the chamber, the vapor is assumed to move adiabatically from the sublimation surface to the condenser. Based on a maximum chamber pressure target of 10 Pa and average product mass transport resistance, the partial pressure at the sublimating interface is estimated to be between 15 and 25 Pa. Using an intermediate pressure corresponds to an equilibrium temperature of $-37°$C, which was used for all simulations.

At the low system pressure, the Reynolds number indicates that the gas flow remains laminar at speeds over 1000 m/s, much higher than the speed of sound. Thus, laminar flow equations are used to model fluid flow.

This mean free path is approximately 1 mm at the target pressure of 10 Pa. At pressures as low as 1 Pa, which may be seen near the condenser, the mean free path approaches 10 mm. The vacuum equipment used has characteristic lengths ranging between 10 cm and

1 m, resulting in a Knudsen number between 10^{-1} and 10^{-3}. This range includes both slip flow and continuum regimes, and the Navier-Stokes equations for compressible gases are appropriate. Because prior work has shown that the slip flow condition does not produce relevant variations for large clearances on both velocity and pressure fields,[1] this condition is not employed to reduce complexity and computation time. It is worth noting that slip effects may become non-negligible as Knudsen number approaches the slip-flow regime. This situation could arise at pressures lower than those primarily analyzed here or in components with significantly smaller hydraulic diameters. In such cases, velocity slip at the wall would reduce shear stress and lead to a slight decrease in predicted pressure drop compared to the no-slip solution.

Conductance Expressions

Table S1 reports the expressions for the conductance of common hardware used in vacuum equipment, which can be found in Barron.[2]

Table S1: Summary of conductance expressions from Barron[2]

**Geometry**	**Equation**
Long Tube	$C = \frac{\pi D^4 \bar{P}}{128\eta L}$
Short Tube	$C = \frac{\pi D^4 \bar{P}}{128\eta L}\left(1 + \frac{0.1427\dot{m}}{\pi\eta L}\right)^{-1}$
90° Elbow	$\frac{\pi K D^3 \bar{P}}{128\eta}$

Detailed Description of Simulated Components

The individual vacuum components (I–IV) and compound assemblies (V) shown in Figure S1 were simulated at the parameter variables shown in Table S2. The straight pipes were

simulated as if they were directly connected to a condenser, so their outlet boundary condition is 0 Pa. The elbows were simulated at various outlet pressures because they were used upstream of the condenser attachment in the vacuum tunnel assembly discussed in **Prediction of Pressure**. The compound assemblies were used to understand the sensitivity of the manifold design to geometrical choices made available when using this topology.

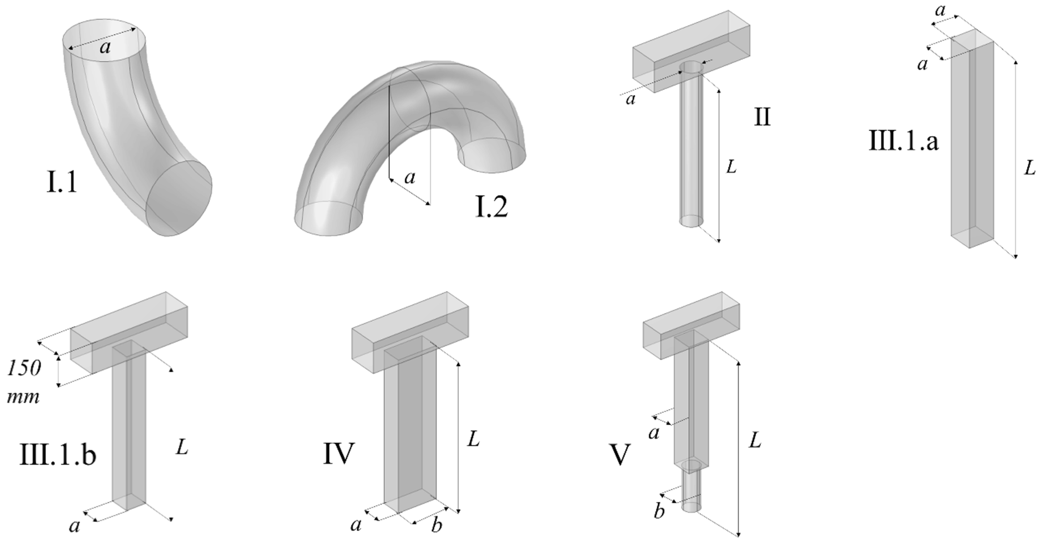

Figure S1: Vacuum element geometries simulated for this work.

Table S2: Vacuum elements and the associated parameters simulated for this work.

Group	ID	Section shape	L, mm	a, mm	b, mm	θ, °	$\dot{m}$, g/h	P_{out}, Pa
I	1	Circular (Bend)	–	100	–	90	27–90	0–10
	2		–	100	–	180	27–90	0–10
II	1	Circular (Straight)	500	40–100	–	–	4.5–150	0
	2		1000	40–100	–	–	4.5–150	0
	3		750	100	–	–	18–90	0
	4		750	160	–	–	36–180	0
III	1	Square	750	100	–	–	18–90	0
	2		750	125	–	–	36–180	0
IV	1	Rectangular	750	100	200	–	36–180	0
V	1	Mixed (square + circular)	750 (500+250)	125	100	–	36–180	0
	2		1000 (750+250)	125	100	–	36–180	0

Effect of Butterfly Valve

While this work uses 90° angle valves to separate the condensers from the vacuum chambers, butterfly valves represent an alternative, in-line valve option. This type of valve was simulated in different positions along a straight pipe to estimate how different flow conditions might affect the pressure drop across the valve. The pipe had a length of 750 mm, and the valve was placed at 1/3, 1/2, and 2/3 of the total pipe length. At the outlet of the pipe the pressure boundary condition was set as $P_{out} = 0$ Pa, while at the inlet the mass flow was varied in the range 18–90 g/h. While including the valve did lead to a small penalty to the pressure drop along the pipe (Figure S2a), the location of the valve did not change this penalty (Figure S2b).

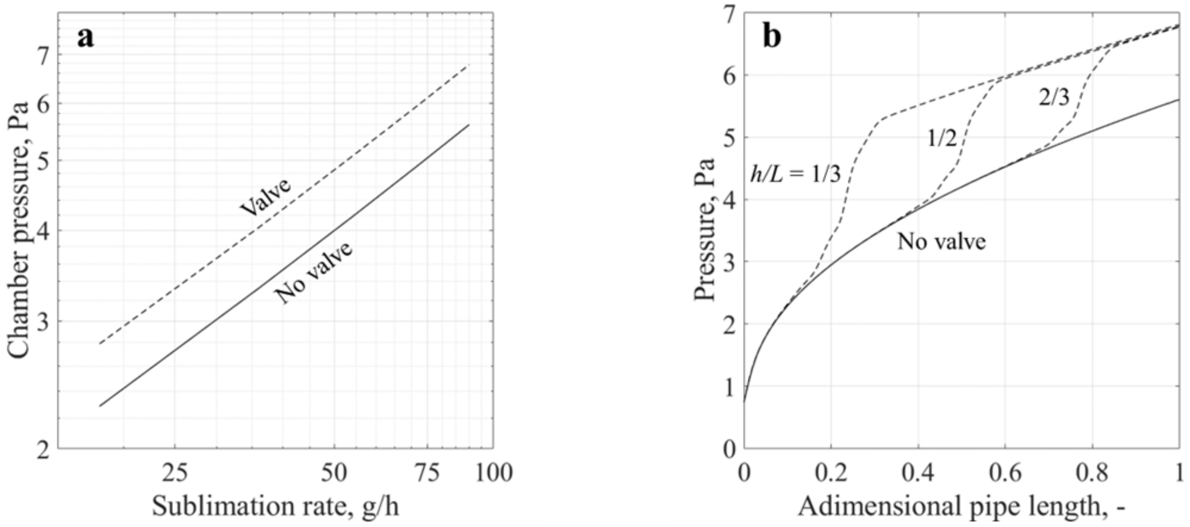

(a) (b)

Figure S2: (a) Penalty associated with adding a butterfly valve to a straight pipe. (b) Plot showing that this penalty is constant irrespective of where the valve is installed.

Single Chamber Experiments

Expected Pressure Variation within the Chamber

The explicit CFD simulations of the single chamber setup also provide a pressure field within the chamber. This pressure is highly uniform with $\Delta P < 0.25$ Pa across the entire volume as shown in Figure S3.

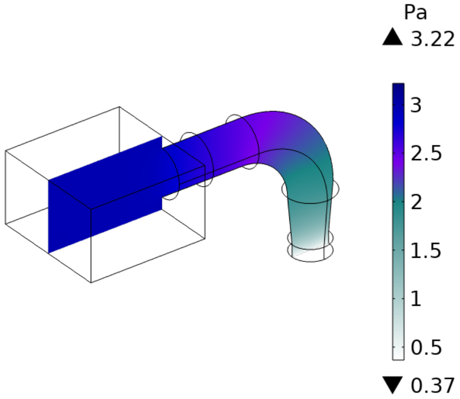

Figure S3: Explicit pressure field from COMSOL within the single vacuum chamber.

Drift in Condenser Level

If the liquid nitrogen level inside the condenser changes during drying, due to factors such as consumption for water vapor condensation or evaporation from thermal losses, the thermal profile of the condenser varies accordingly, causing the position where the water vapor condenses to move with it. To evaluate the influence of this effect on the final pressure inside the drying chamber, varying fill heights of the condenser were simulated. Specifically, the length of the cylinder after the reducer in Figure 2b was varied between 50 mm (full condenser) and 300 mm (almost empty condenser). For a given mass flow, it is possible to obtain a relationship between the level of the liquid nitrogen in the condenser and the pressure inside the chamber. Then, by estimating the level dynamics, it is possible to measure the pressure dynamic during an experiment and check their correspondence.

The power delivered to the liquid nitrogen, PW_{LN_2}, through condensation of water vapor and heat losses through the dewar can be estimated from

$$PW_{LN_2} = \dot{m}_w \Delta H_{cond,w} + PW_{loss} \tag{4}$$

where $\Delta H_{cond,w}$ is the latent heat of condensation of the water vapor. The level, z, inside the condenser changes according to

$$\rho_{LN_2} S \frac{dz}{dt} = \frac{PW_{LN_2}}{\Delta H_{v,LN_2}} \tag{5}$$

where ρ_{LN_2} is the density of liquid nitrogen, $\Delta H_{v,LN_2}$ is the latent heat of evaporation of liquid nitrogen, and S is the free surface of the liquid nitrogen. By using Equation 5, the time evolution of the condenser level was calculated. Combining this level evolution with the simulated relationship between condenser level and chamber pressure then provided for the time evolution of the pressure inside the chamber for a given mass flow rate of water vapor. The experimental results for the small vacuum chamber confirm this behavior, obtained through a gravimetric test without refilling the condenser. The chamber pressure trace compares extremely well to the simulated value of 2.1 mm/min calculated using the aforementioned procedure.

Vial Holder for Sublimation Experiments

The acrylic vial holder (Figure S4a) was laser cut from two sheets of acrylic to hold the vials within the small vacuum chamber. This holder fits within the internal volume of the chamber while separating the vials from the chamber floor to limit heat transfer without the need for temperature control of the shelf (Figure S4b).

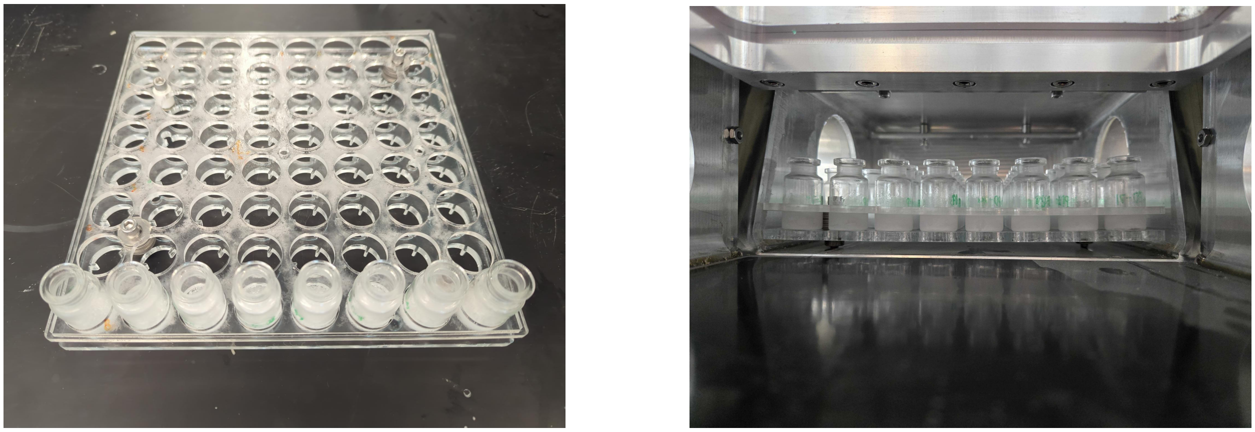

(a) (b)

Figure S4: (a) The acrylic tray used to offset the vials from the chamber floor. (b) The tray in the chamber.

Condenser Design

The condensers used in this work consist of a stainless steel inner body and an outer liquid nitrogen dewar. The stainless steel body is made of tubing with an ISO claw-clamp fitting welded on one end and a cap welded on the other end. A half-nipple is welded on the side of the condenser body near the open side for the other condenser port. An outer collar on the condenser body allows it to rest on top of the dewar lid, suspending the condenser body inside the dewar. The dewar lid includes holes for liquid nitrogen refilling along with high- and low-level thermocouple sensors to enable automated filling.

During initial operation, the condenser did not effectively condense much water vapor, as the condenser body walls between the condenser ports were both too short and too warm to effectively condense water vapor before it exited the condenser. Thus, an inner tube was added to the condenser to force the water vapor to travel through the full length of the condenser body before exiting. This tube significantly improved condenser performance. Although the addition of the inner tube introduces an extra internal element, its impact on the overall pressure balance is negligible under the operating conditions considered in this study. The amount of non-condensable gases present in the system is extremely limited and

remains well within the pumping capacity, preventing any significant pressure build-up inside the condenser. Furthermore, due to the low temperature of the condenser surfaces, water vapor condenses immediately upon contact and does not propagate to the lower section of the condenser or the downstream portion of the inner tube. As a result, the added internal tube primarily improves condensation efficiency without introducing a measurable additional pressure drop.

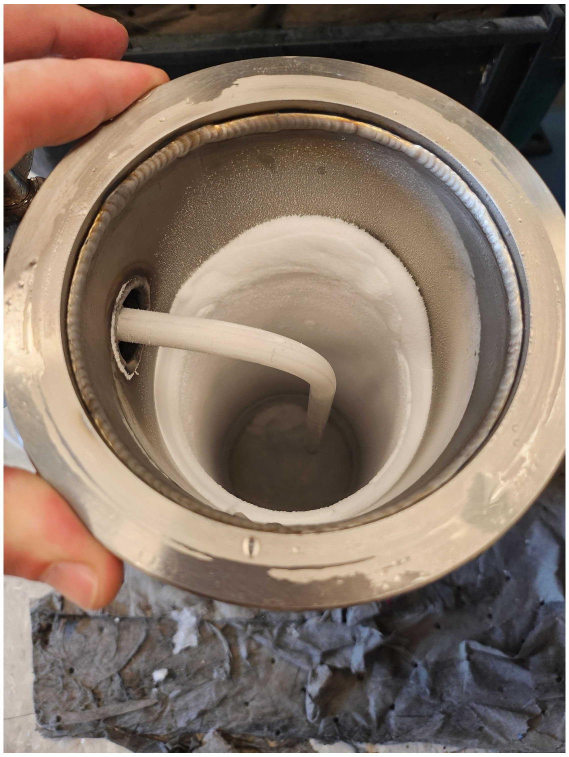

Figure S5: Image of the internal geometry of the ISO100 sized condensers used on the large vacuum tunnel.

Tunnel Simulation Details

Manifold Simulation Results

Figure S6b shows the midline pressure trace, along with the inlet and outlet pressures, of the simulated manifold along its length. The outlet port pressure was determined by simulation of a 90° radius elbow connected directly to a condenser. The manifold produces a relatively

small pressure drop of around 0.3 Pa. Additionally, the pressure along the various inlet ports is highly uniform, as the maximum variation in pressure between inlet ports is less than 0.1 Pa.

Such low pressure drops can be attributed to the fact that the manifold acts as an expansion chamber in which the various vapor streams can merge with minimal viscous stresses. Thus, the manifold design is a viable option when a multitude of vacuum ports from the chamber need to be connected to a small number of condensers (or vice versa).

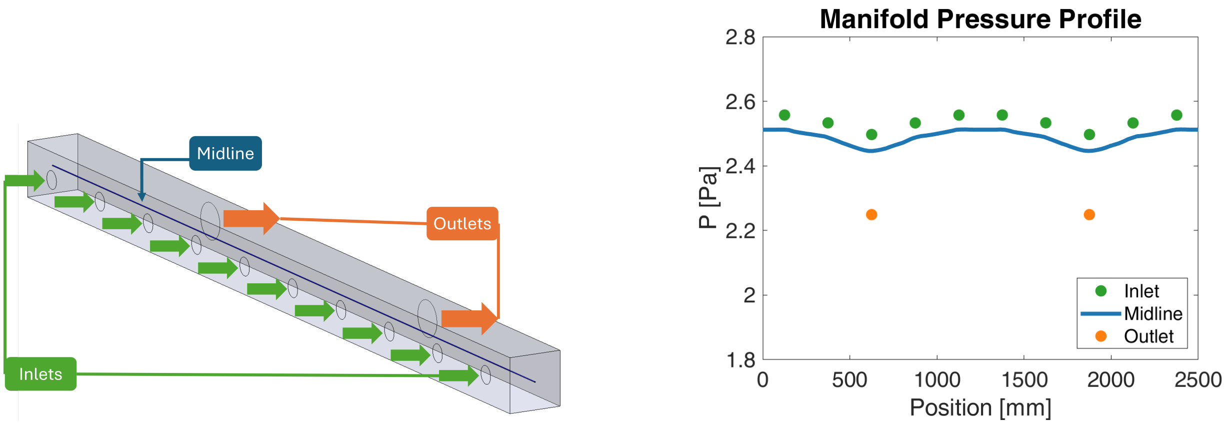

(a) (b)

Figure S6: (a) Schematic for how the manifold simulations were run. (b) Pressure profile along the long dimension.

Accuracy of Component Concatenation

For the direct attachment simulation, the tunnel volume was simulated both with and without explicitly including the ISO100 90° radius elbow geometry. When the tunnel was simulated without the radius elbow, the upstream pressure value from the radius elbow simulation with a boundary condition of $P = 0$ Pa was used as the outlet boundary condition for the tunnel ports. When the radius elbow was explicitly included in the simulated geometry, the boundary condition of $P = 0$ Pa was applied at the elbow outlet. The results of these simulations match extremely well, further validating the concatenation method for pressure prediction.

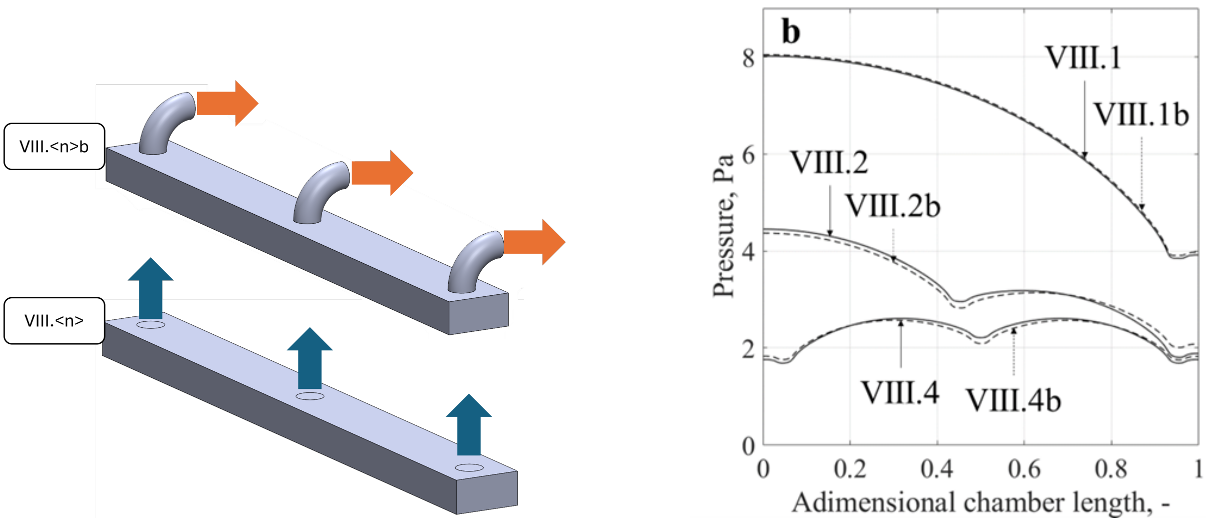

(a) (b)

Figure S7: (a) The direct attachment simulations were calculated both by explicitly including the radius elbow geometry and by using the upstream pressure calculated for a radius elbow as the boundary condition for the tunnel. (b) Comparison between the simplified simulations (solid lines) and simulations where the outlet was simulated as an ISO100 90° elbow (dashed lines). The legend of the connections refers to Figure 5a.

When explicitly computing the ISO100 90° elbows, as depicted in Figure S7b, the results slightly differed due to a partial redistribution of mass flow rates between the various ports (shown by dashed lines). This redistribution was influenced by the fact that ports handling higher mass flow rates experienced greater pressure drops, causing part of the vapor they evacuated to migrate toward ports with lower pressures, partially equalizing the situation. However, the difference between the simplified and explicit simulations never exceeded 0.2 Pa, which is acceptable considering the reduction in complexity achieved by the concatenation of the two simulations.

Tunnel Experiments

The tunnel simulation results were validated by performing several experiments with a varying sublimation flux load in the constructed system described in **Prediction of Pressure**.

This load was generated by placing between one and twenty four 50 mL falcon tubes each filled with 10 mL of a 5% mannitol solution on four magnetically levitated trays (Figure S8) and allowing them to sublimate for 2 hours.

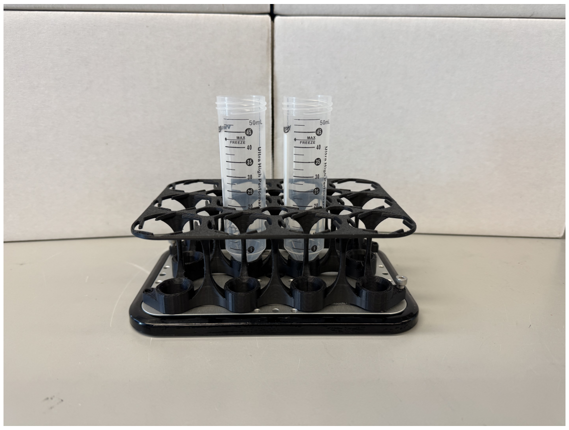

Figure S8: Image of the Falcon tube holder used to artificially generate sublimation loads.

In this implementation, the locations of the condensers were limited by other nearby vacuum components and were chosen to maximize the distance between them while allowing for the installation of a third condenser. Additionally, to generate a uniform flux across the tunnel (as was assumed in the simulation), the four trays were evenly distributed across the tunnel, with falcon tubes distributed evenly among the trays. Falcon tubes were employed in the study to ensure higher filling volume for the same exposed freezing surface area. Therefore, falcon tubes allow for a thicker ice layer that extends drying duration, thus improving measurability and validation conditions. To measure the pressure distribution within the tunnel, five pressure gauges (three Pirani and two diaphragm) were used and attached to different tunnel modules. A schematic view of this experimental setup is shown in Figure S9, representing the tunnel described in **Continuous Lyophilizer Tunnel Validation**.

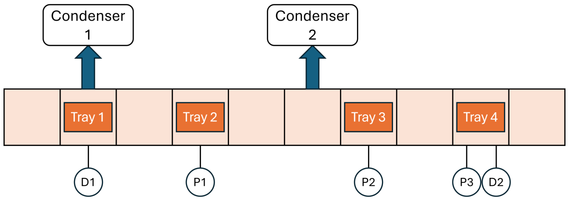

Figure S9: Schematic view of the vacuum chamber showing condenser attachment locations, tray positions, Pirani gauge locations (P#), and diaphragm gauge locations (D#).

References

(1) Barresi, A. A.; Marchisio, D. L. Computational Fluid Dynamics data for improving freeze-dryers design. *Data in Brief* **2018**, *19*, 1181–1213.

(2) Barron, R. F. *Cryogenic systems*, 2nd ed.; Monographs in cryogenics 3; Oxford Univ. Press: New York, 1985.
